# A Novel Image Encryption Algorithm Based on DNA Subsequence Operation

**DOI:** 10.1100/2012/286741

**Published:** 2012-10-11

**Authors:** Qiang Zhang, Xianglian Xue, Xiaopeng Wei

**Affiliations:** Key Laboratory of Advanced Design and Intelligent Computing, Ministry of Education of Dalian University, Dalian 116622, China

## Abstract

We present a novel image encryption algorithm based on DNA subsequence operation. Different from the traditional DNA encryption methods, our algorithm does not use complex biological operation but just uses the idea of DNA subsequence operations (such as elongation operation, truncation operation, deletion operation, etc.) combining with the logistic chaotic map to scramble the location and the value of pixel points from the image. The experimental results and security analysis show that the proposed algorithm is easy to be implemented, can get good encryption effect, has a wide secret key's space, strong sensitivity to secret key, and has the abilities of resisting exhaustive attack and statistic attack.

## 1. Introduction

Nowadays, the computer network has changed the mode of people's communication. People can easily transfer the various multimedia information through the network. However, because of the openness of the network, people have to take more and more attention on security and confidentiality of multimedia information. The digital image is an important information vector of multimedia communication, thus how to protect the image information becomes a universal concern problem for people. The traditional image encryption methods (such as DES, IDEA, and AES) are not suitable for image encryption due to the different storage format of an image. The new research algorithms of image encryption are needed urgently.

As an example, due to chaotic system with like-random, high sensitivity to initial value, and unforeseeable properties, chaos based cryptosystems have become current research hotspot. According to the object of scrambling, the chaos-based algorithms operate in two stages: the shuffling stage and the substitution stage. In the shuffling stage, the position of pixels from original image is changed by chaotic sequences [1] or by some matrix transformation, such as Arnold transform, magic square transform, and so forth. These shuffling algorithms are easy to be realized and have better encryption effect. Due to these shuffling algorithms, just changing the position of pixels but not changing the pixel values leads to the histogram of the encryption image is the same as the original image, and thus its security is threatened by the statistical analysis. In the substitution stage, the pixel values are changed by chaotic sequences. Most of these encryption methods are directly implemented by overlaying a chaotic sequence generated by a single chaotic map and the pixel grey value from the image. Comparing with the method of the shuffling, the method of value substitution may lead to higher security, but from the vision angle the encryption effect is not good. Thereby, in order to improve the security and the encryption effect, the shuffling and the value substitution are combined by some researchers, the readers can refer to [2, 3]. However, only using a single chaotic map to encrypt image may result in lower security and smaller key space. Ren et al. [1] presented a chaotic algorithm of image encryption based on dispersion sampling; their algorithm has better scrambling effect, but has small key space. Zhang et al. [4] use logistic and standard systems to scramble the location and the value of pixel points from the image, and they have got better result; however, no security analysis in their paper was given. Recently, Lian et al. [5–7] used some multidimensional chaotic maps (such as the chaotic standard map, the chaotic neural networks, and spatiotemporal chaos system) to encrypt images and make a detailed analysis for the security of algorithms. Their algorithms have satisfactory security with a low cost. A new image encryption algorithm based on multiple chaos system is proposed by Zuo et al. [8]. Similarly, Liu et al. [9] also used multiple chaotic maps to encrypt image. All of their algorithms have a large key space, high sensitivity to key variation, and unforeseeable and have the ability of resisting traditional attacks. Generally speaking, to improve the security of encryption algorithm, researchers usually try to use more complex chaotic system or combine some new encryption methods with the existing chaotic systems to implement image encryption. However, some chaotic systems have been proven to be insecure [10–12]. 

With the rapid development of DNA computing, DNA cryptography, as a new field, has come into being. A method for hiding message in DNA microdots was proposed by Clelland et al. [13]. Clelland used DNA microdots to hide message to implement the protection of information. For instance, letter *A* is expressed as DNA sequence *GGT* by complex biological operation. Obviously, it is difficult to be implemented and is not suitable for image encryption. Gehani et al. presented an encryption algorithm of the one-time pad cryptography with DNA strands [14]; Gehani's method is effective, but the process of encryption must utilize complex biological operations, which are difficult to be controlled under the experimental environment. So the method is not easy to be realized. In fact, since the high-tech laboratory requirements and computational limitations, combining with the labor intensive extrapolation means, researches of DNA cryptography are still much more theoretical than practical. Recently, Kang presented a pseudo DNA cryptography method [15]. Kang's method not only has the better encryption effect, but also does not require complex biological operation. However, it was only used for encrypting text files.

In this paper, we do not use biological operation to implement image encryption, but adopt the rule of DNA subsequence operation such as truncation operation, deletion operation, transformation operation and so forth, then combine DNA subsequence operation with chaos system to scramble the location and the value of pixel point from the image.

The structure of this paper is as follows. In Section 2, we will introduce the basic theory of the proposed algorithm. The design of the proposed image encryption scheme is discussed in the Section 3. In Section 4, some simulation results and security analysis are given. In Section 5, we compare our algorithm with other encryption algorithms.  Section 6 draws the conclusion. 

## 2. Basic Theory of the Proposed Algorithm

### 2.1. Generation of the Chaotic Sequences

 The chaotic system is a deterministic nonlinear system. It possesses a varied characteristics, such as high sensitivity to initial conditions and system parameters, random-like behaviors, and so forth. Chaotic sequences produced by chaotic maps are pseudo-random sequences; their structures are very complex and difficult to be analyzed and predicted. In other words, chaotic systems can improve the security of encryption systems. Thus, it is advisable to encrypt digital image with chaotic systems [16–24]. Here, we introduce the following two chaotic maps, one is logistic map, and the other is 2D logistic map. In the paper, we use 2D logistic map to produce the eight parameters as the initial values and system parameters of four logistic maps.

Logistic map is an example for chaotic map, and it is described as follows:

(1) $$\begin{array}{c} x_{n+1}=\mu x_{n}\left(1-x_{n}\right), \end{array}$$

where *μ* ∈ [0,4], *x*_*n*_ ∈ (0,1), and *n* = 0,1, 2,…. The research result shows that the system is in chaotic state under the condition that 3.56994 < *μ* ≤ 4.

2D logistic map is described in ([Disp-formula EEq2]) [25] as follows:

(2) xi+1=μ1xi(1−xi)+γ1yi2yi+1=μ2yi(1−yi)+γ2(xi2+xiyi).

When 2.75 < *μ*_1_ ≤ 3.4, 2.75 < *μ*_2_ ≤ 3.45, 0.15 < *γ*_1_ ≤ 0.21 and, 0.13 < *γ*_2_ ≤ 0.15, the system is in chaotic state and can generate two chaotic sequences in the region (0,1]. Due to the system parameter *γ*_1_ and *γ*_2_ which have smaller value range, we set *γ*_1_ = 0.17 and *γ*_2_ = 0.14, other parameters can be seen as secret keys. 

### 2.2. DNA Sequence Encryption

#### 2.2.1. DNA Encoding and Decoding for Image

 A single DNA sequence is made up of four nucleic acid bases: *A* (adenine), *C* (cytosine), *G* (guanine), and *T* (thymine), where *A* and *T* are complements, and *C* and *G* are complements. Let binary number 0 and 1 be complements, so 00 and 11 are complements, and 01 and 10 are complements. Thus we can use these four bases: *A*, *T*, *G*, and *C* to encode 01, 10, 00, and 11, respectively. The encoding method still satisfies the Watson-Crick complement rule [25]. Usually, each pixel value of the 8 bit grey image can be expressed to 8 bits binary stream. The binary stream can be encoded to a DNA sequence whose length is 4. For example: if the first pixel value of the original image is 75, convert it into a binary stream [01001011]. By using the above DNA encoding rule to encode the stream, we can get a DNA sequence [*AGTC*], whereas we use *A*, *T*, *G*, and *C* to express 01, 10, 00, and 11, respectively. We can get a binary sequence [01001011].

#### 2.2.2. DNA Subsequences Operation

 In this section we use the idea of [26] to define the DNA subsequence and the corresponding operation. We define that a DNA sequence *P*_*k*_ contains *m* strands of DNA subsequences according to the order, in the *P*_*k*_, the number of bases is *k* (*m* ≤ *k*). The expression is *P*_*k*_ = *P*_*m*_ *P*_*m*−1_ ⋯ *P*_2_ *P*_1_. The number of bases for the corresponding DNA subsequences is *l*_*m*_ *l*_*m*−1_ ⋯ *l*_2_ *l*_1_, respectively. Apparently, *k* = *l*_*m*_ + *l*_*m*−1_ + ⋯*l*_2_ + *l*_1_. Based on the above DNA subsequence expression, we described the following five kinds of DNA subsequence operation; they are elongation operation, truncation operation, deletion operation, insertion operation, and transformation operation.

1. DNA subsequence elongation operation.

**Definition 1**

We suppose that there is an original DNA sequence *P*_1_, the subsequence *P*_2_, whose length is *l*_1_, is elongated to the tail of *P*_1_. After elongation operation, we can get a new DNA sequence *P*′ = *P*_1_ *P*_2_. The expression is as follows:

(3) $$\begin{array}{c} P_{1}+P_{2}\to P_{1}P_{2}. \end{array}$$

(2) DNA subsequence truncation operation.

**Definition 2**

The truncation operation and the elongation operation are contrary. Truncating the end of the subsequence *P*_2_ in the DNA sequence *P*_1_ *P*_2_, we will obtain a new DNA sequence *P*′ = *P*_1_. The expression is as follows:

(4) $$\begin{array}{c} P_{1}P_{2}-P_{2}\to P_{1}. \end{array}$$

(3) DNA subsequence deletion operation.

**Definition 3**

We suppose that there is an original DNA sequence *P* = *P*_3_ *P*_2_ *P*_1_. Deleting the subsequence *P*_2_, then we will obtain a new DNA sequence *P*′ = *P*_1_ *P*_3_. The expression is as follows:

(5) $$\begin{array}{c} P_{3}P_{2}P_{1}-P_{2}\to P_{3}P_{1}. \end{array}$$

(4) DNA subsequence insertion operation.

**Definition 4**

The deletion operation and the insertion operation are contrary. We suppose that there is an original DNA sequence *P* = *P*_3_ *P*_1_, inserting a subsequence *P*_2_, whose length is *l*_2_, into *P*. The expression is as follows:

(6) $$\begin{array}{c} P_{3}P_{1}+P_{2}\to P_{3}P_{2}P_{1}. \end{array}$$

(5) DNA subsequence transformation operation.

**Definition 5**

In brief, the locations of two subsequences are transformed. If the original DNA sequence is *P* = *P*_5_ *P*_4_ *P*_3_ *P*_2_ *P*_1_. Transforming the locations of *P*_4_ and *P*_2_, we will get a new DNA sequence *P*′ = *P*_5_ *P*_2_ *P*_3_ *P*_4_ *P*_1_. The expression is as follows:

(7) $$\begin{array}{c} P_{5}P_{4}P_{3}P_{2}P_{1}\to P_{5}P_{2}P_{3}P_{4}P_{1}. \end{array}$$

We introduced five kinds of DNA subsequence operations, where the inverse operation of elongation operation is truncation operation and the inverse operation of deletion operation is insertion operation. In our algorithm, we use elongation operation, truncation operation, deletion operation, and transformation operation and combined with the use of the Logistic chaotic map we will realize the image encryption algorithm. However, the insertion operation is just used in the decryption process.

## 3. Algorithm Description

### 3.1. Generation of Chaotic Sequences

 Input initial state (*x*_0_, *μ*_1_, *y*_0_, *μ*_2_), by using 2D Logistic to produce eight parameters (*x*_1_, *x*_2_, *x*_3_, *x*_4_, *x*_5_, *x*_6_, *x*_7_, *x*_8_) after iterating 1000 times. We Use the following formulas to produce four groups of parameters:

(8) x1=x1,  u1=3.9+0.1×x2,y1=x3,  u2=3.9+0.1×x4,z1=x5,  u3=3.9+0.1×x6,q1=x7,  u4=3.9+0.1×x8.

Then, by using logistic chaotic map to generate four chaotic sequences under the condition that the four groups of initial values are (*x*_1_, *u*_1_), (*y*_1_, *u*_2_), (*z*_1_, *u*_3_), and (*q*_1_, *u*_4_), their length are *m* × *n*, respectively. 

### 3.2. Generation of DNA Subsequences

**Step 1**

Input an 8 bit grey image *A*(*m*, *n*), as the original image, where *m* and *n* is rows and columns of the image.

**Step 2**

Convert image *A* into binary matrix *A*′ whose size is (*m*, *n* × 8) and divide *A*′ into eight bit-planes. Here, the first bitplanes and the eighth bitplanes, the second bitplanes and the seventh bitplanes, the third bitplanes and the sixth bitplanes, and the forth bit-planes and the fifth bit-planes are composed, respectively. Then we obtain four bit-planes.

**Step 3**

Carry out DNA encoding operation according to Section 2.2.1 for the four bitplanes, then we get four coding matrices *P*_1_, *P*_2_, *P*_3_, *P*_4_, all of their sizes are (*m*, *n*).

**Step 4**

Convert *P*_1_, *P*_2_, *P*_3_, *P*_4_ into *P*_1_′, *P*_2_′, *P*_3_′, *P*_4_′ whose sizes are (1, (*m* × *n*)), then divide *P*_1_′, *P*_2_′, *P*_3_′, *P*_4_′ into DNA subsequence; the average length of subsequences are *l*_1_ = 128, *l*_2_ = 64, *l*_3_ = 32, and *l*_4_ = 8, respectively. So, there are the following conclusions:

(9) P1′=p11p12⋯p1(mn/l1),P2′=p21p22⋯p2(mn/l2),P3′=p31p32⋯p3(mn/l3),P4′=p41p42⋯p4(mn/l4),

where *p*_*ij*_ are DNA subsequences, *l*_*i*_ are lengths of these subsequences, *i* ∈ [1,4], and *j* ∈ [1, *mn*/*l*_*i*_].

### 3.3. Deletion Operation

**Step 1**

We suppose that there is a chaotic sequence *X* = {*x*_1_, *x*_2_ ⋯ *x*_*mn*/*l*_*i*__}.

**Step 2**

If *x*_*i*_ < 0.5, delete the *i*th subsequence according to Section 2.2.2, otherwise save the subsequence.

**Step 3**

Those deleted subsequences are moved to the end of the saved subsequences.

### 3.4. Transformation Operation

**Step 1**

We suppose that there is a chaotic sequence *X* = {*x*_1_, *x*_2_ ⋯ *x*_*mn*/*l*_*i*__}.

**Step 2**

To sort *X* by ascending, we get a new sequence *X*′ = {*x*_1_′, *x*_2_′ ⋯ *x*_*mn*/*l*_*i*__′}.

**Step 3**

If *x*_*i*_ < 0.5, the *i*th subsequence and the *i*′th subsequence from the location of *X*′ are transformed according to Section 2.2.2.

### 3.5. Elongation and Truncation Operation

**Figure 1:**
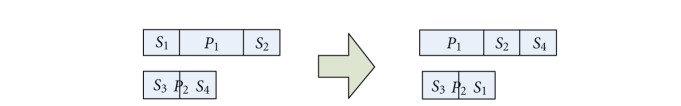
Elongation and truncation operation of DNA subsequences.

As shown in Figure 1, *P*_1_ and *P*_2_ are two DNA subsequences from any of two bit-planes, we suppose that the length of *P*_1_ is 128, the length of *P*_2_ is 64, *S*_1_ and *S*_2_, *S*_3_, and *S*_4_ are DNA subsequences of *P*_1_ and *P*_2_, respectively. First, we truncate *S*_1_ and *S*_4_, then elongate *S*_1_ to the tail of *P*_2_, elongate *S*_4_ to the tail of *P*_1_.

### 3.6. Complement Operation

 Complement operation is carried out for every one dimension bit-plane whose size is (1, *m* × *n*), we suppose there is a chaotic sequence *X* = {*x*_1_, *x*_2_ ⋯ *x*_*mn*/*l*_*i*__}. If *x*_*i*_ < 0.5, the nucleic acid base of the *i*th location is complemented, otherwise, it is unchanged. 

### 3.7. The Procedure of Image Encryption and Decryption

**Figure 2:**
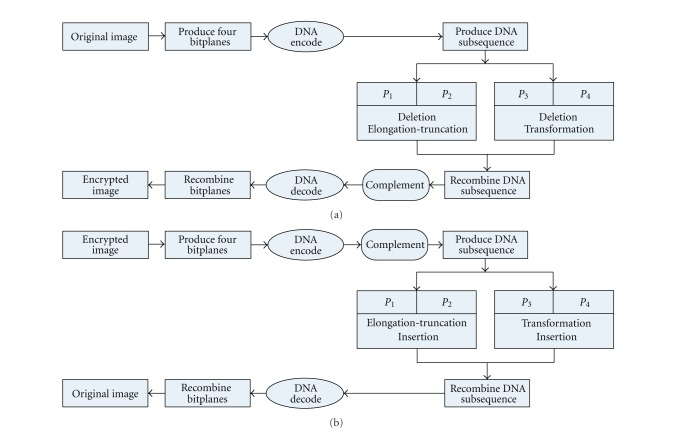
The block diagram of the proposed algorithm. (a) The block diagram of the encryption algorithm. (b) The block diagram of the decryption algorithm.

 The proposed encryption algorithm includes three steps: first, by using the method proposed in the Section 3.1 to produce four groups of DNA sequences *P*_1_, *P*_2_, *P*_3_, and *P*_4_, where *P*_*i*_  (*i* = 1,2, 3,4) is made up of many DNA subsequences. Then, to disturb the position and the value of pixel points from image by combining the logistic map, generate chaotic sequences and DNA subsequence operations (such as elongation operation, truncation operation, deletion operation, transformation, etc.). At last, the encrypted image is obtained by DNA decoding and recombining bit-planes. The block diagram of the proposed algorithm is shown in Figure 2, the block diagram of the encryption algorithm is shown in Figure 2(a), and Figure 2(b) shows the block diagram of the decryption algorithm. We can see that the procedure of image decryption is inverse procedure of image encryption from Figure 2. The detailed procedure of our encryption and decryption algorithms are explained in the following pseudo-codes (Algorithms 1 and 2).

The functions and parameters in Algorithms 1 and 2 are the same as Sections 3.1–3.6, where DNA decoding and recombining are the inverse process of Steps 3 and 2 in Section 3.2. The procedure of acquiring the original image from the encryption image is an inverse operation according to Algorithm 2, where deletion operation is replaced by insertion operation.

## 4. Simulation Result and Security Analysis

### 4.1. Simulation Result

**Figure 3:**
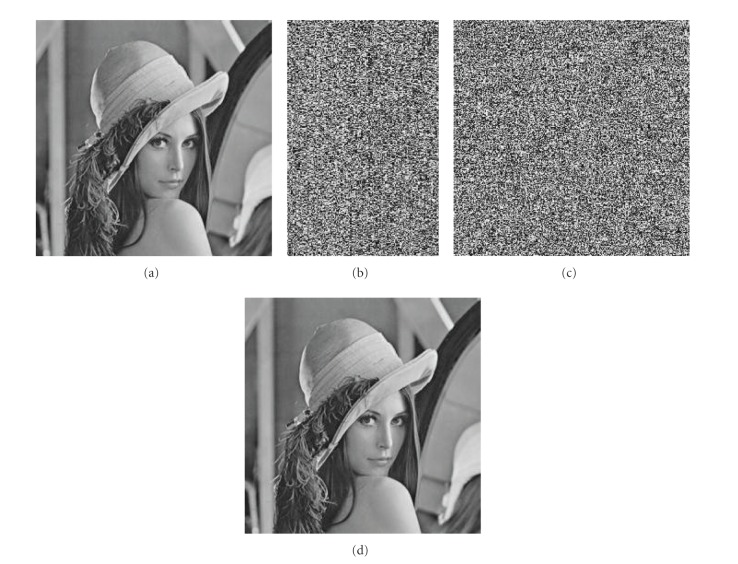
Encrypted image and decrypted image. (a) The original image. (b) The encrypted image. (c) The decrypted image under the wrong secret keys. (d) The decrypted image under the correct secret keys.

 In this paper, for standard 256 × 256 gray image Lena, we use Matlab 7.1 to simulate experiment. In our experiment, we set *x*_0_ = 0.95, *μ*_1_ = 3.2, *γ*_1_ = 0.17, *y*_0_ = 0.25, *μ*_1_ = 3.3, *γ*_2_ = 0.14. The original image is shown in Figure 3(a), Figure 3(b) shows encrypted image, and Figure 3(b) points out that it is difficult to recognize the original image. Figures 3(c) and 3(d) show the decrypted image under the wrong secret keys and the right secret keys, respectively. From Figure 3(c), we know that it has not any connection with the original image, but Figure 3(d) is as same as the original image.

### 4.2. Secret Key's Space Analysis

 In the proposed algorithm, the initial value and the parameter of the system of 2D logistic are identified as secret keys of this algorithm. Therefore, our algorithm has six secret keys *x*_0_, *μ*_1_, *γ*_1_, *y*_0_, *μ*_2_, *γ*_2_. If the precision is 10^−14^, the secret key's space is 10^14^ × 10^14^ × 10^14^ × 10^14^ × 10^14^ × 10^14^ = 10^84^ ≈ 2^279^. It is shown that the secret key's space is large enough to resist exhaustive attack.

### 4.3. Secret Key's Sensitivity Analysis

**Figure 4:**
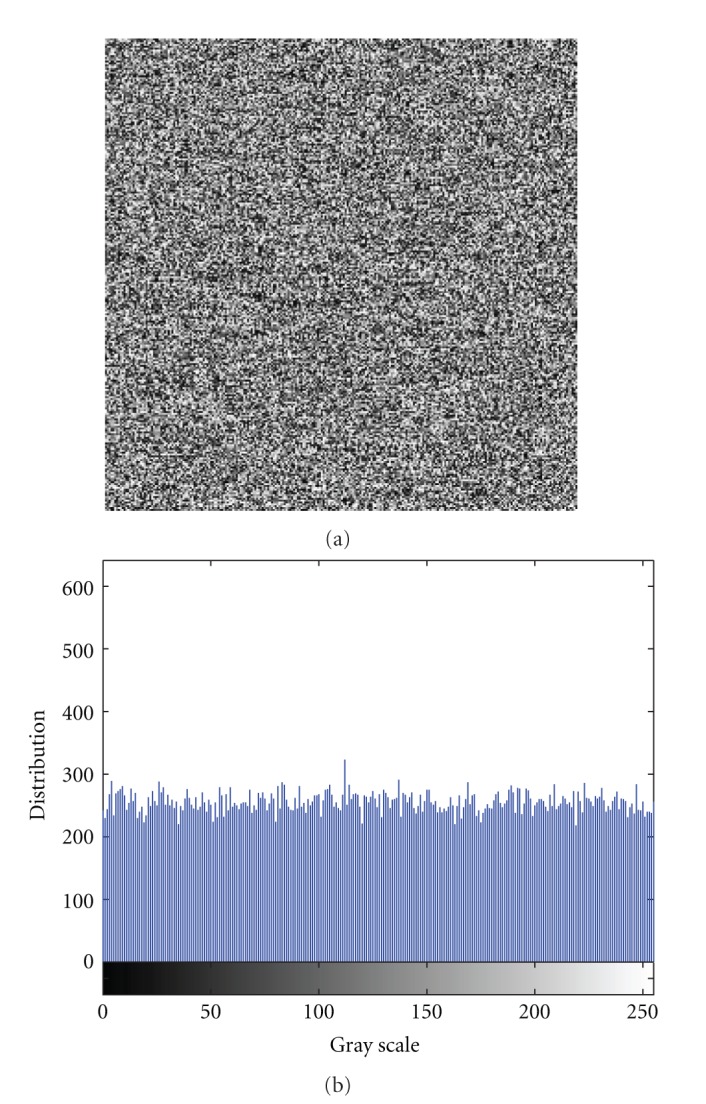
The sensitivity of secret key *x*_0_. (a) The decrypted image with secret key (0.95000000000001,3.2,0.17,0.25,3.3,0.14). (b) The corresponding histogram.

 The chaotic map is very sensitive to the initial value in chaotic state, in other words, it also ensured the sensibility of this encryption algorithm to the secret key. In this paper, if the initial values from three chaotic maps are changed a little, the recovering image is not allowed to be read, but we can get the original image from the encrypted image by using the correct secret keys. The experiment results are shown in Figure 4, where Figure 4(a) shows the decrypted image under the secret keys (0.95000000000001,3.2,0.17,0.25,3.3,0.14). The corresponding histogram is shown in Figure 4(b), and we can see that the histogram of the decrypted image is very uniform. The sensitivity of other parameters is similar. From Figure 4, we can see that only when all secret keys (the chaotic initial value and system parameter) are correct, the original image can be obtained. Otherwise the decrypted image will have no connection with the image. Based on the above argument, our algorithm has strong sensitivity to secret key and we can say again that our algorithm can resist exhaustive attack.

### 4.4. Statistical Analysis

#### 4.4.1. The Grey Histogram Analysis

**Figure 5:**
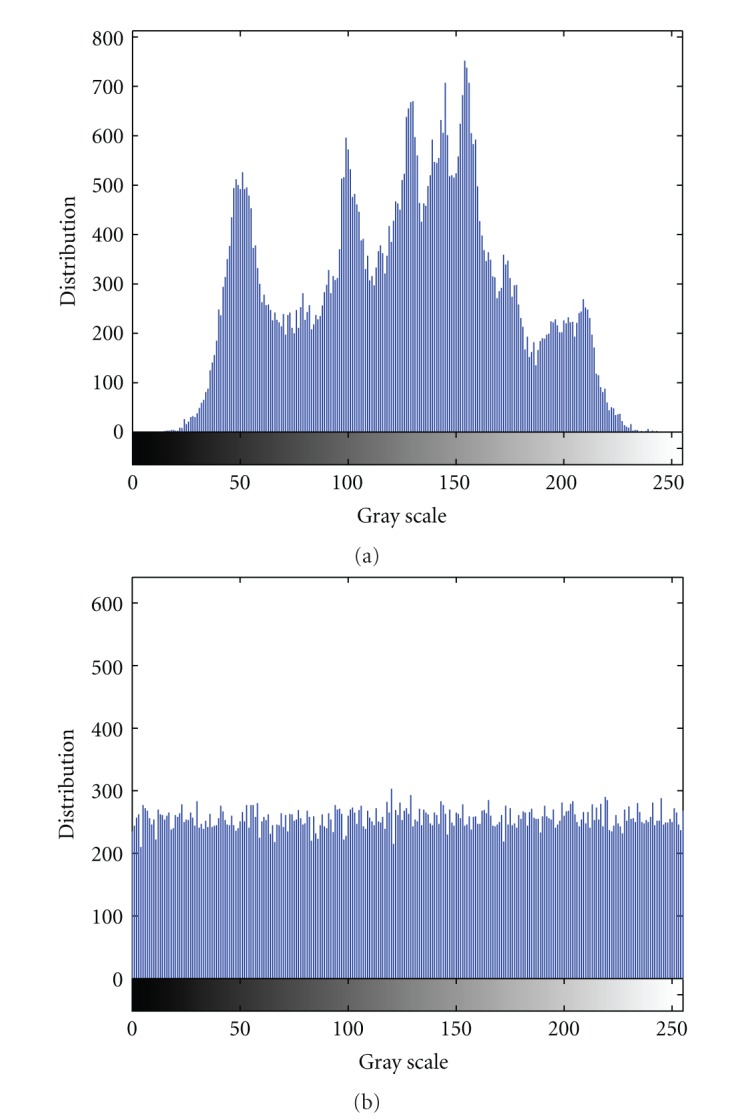
The grey histogram of the original image and the encrypted image. (a) The grey histogram of the original image. (b) The grey histogram of the encrypted image.

 We compare the grey histogram of the image before and after encryption to analyze the statistical performance.  Figure 5(a) shows the grey histogram of the original image and Figure 5(b) shows the grey histogram of the encrypted image. From the two figures, we can see that the original pixel grey values are concentrated on some value, but the pixel grey values after the encryption are scattering in the entire pixel value space, namely, two images have lower similarity. Clearly, it is difficult to use the statistical performance of the pixel grey value to recover the original image. Thereby, our algorithm has strong ability of resisting statistical attack.

#### 4.4.2. Correlation Coefficient Analysis

 The correlation of the adjacent pixels in original image is very high, an effective encryption algorithm can reduce the correlation of between adjacent pixels. Here, we randomly select 3000 pairs (horizontal, vertical and diagonal) of adjacent pixels from the original image and the encrypted image, then by using the following formulas to calculate the correlation coefficient:

(10) E(x)=1N∑i=1Nxi,D(x)=1N∑i=1N(xi−E(x))2,cov⁡(x,y)=1N∑i=1N(xi−E(x))(yi−E(y)),rxy=cov⁡(x,y)D(x)×D(y),

where *x* and *y* are grey value of two adjacent pixels in the image.

**Figure 6:**
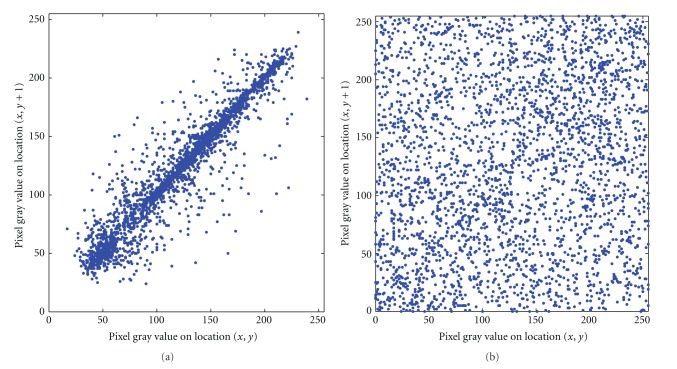
Correlation of two horizontally adjacent pixels in the original image and in the encrypted image.

Figures 6(a) and 6(b) show the correlation of two horizontally adjacent pixels in the original image and that in the encrypted image, where the correlation coefficients are 0.9432 and 0.1366, respectively. Other results are shown in Table 1. From Figure 6(b) and Table 1, we can see that the correlation coefficient of the adjacent pixels in encrypted image is low, which is close to 0. It follows from Figure 6(b) and Table 1 that the proposed image encryption algorithm has strong ability of resisting statistical attack.

#### 4.4.3. Information Entropy

 It is well known that information entropy can measure the distribution of grey value in the image. We can make sure that the bigger information entropy the more uniform for the distribution of grey value. The definition of information entropy is as follows:

(11) $$\begin{array}{c} H\left(m\right)=-\sum_{i=0}^{L}P\left(m_{i}\right)\text{lo}{\text{g}}_{\text{2}}P\left(m_{i}\right), \end{array}$$

where *m*_*i*_ is the *i*th grey value for *L* level grey image and *P*(*m*_*i*_) is the emergence probability of *m*_*i*_. The information entropy of an idea random image is 8. For the proposed algorithm, the information entropy is 7.9975. It is very close to 8. 

## 5. Comparison with Other Encryption Algorithms

 In this section, we will compare our proposed algorithm with existing chaos-based and DNA-based encryption algorithms. We focus on the security consideration in the comparative aspects of chaos-based and focus on the encryption objects and environment in the comparative aspects of DNA-based. The comparison results are shown in Tables 2 and 3. From Table 2, we can see that the key space and the information entropy of our proposed algorithm are larger than others. However the methods in [17, 18] can resist differential attack, the proposed method in this paper cannot resist differential attack. From Table 3, we easily found that only our algorithm and [14] can implement image encryption. But the algorithm proposed in [14] is difficult to be implemented owing to the complex biologic operation. Kang's encryption effect [15] is better than others. However, his algorithm can only encrypt the text. After comparing with other encryption algorithms proposed in Tables 2 and 3, the proposed algorithm is better than other DNA-based encryption algorithm and has larger key space and high key sensitivity, but the disadvantage is that the algorithm cannot resist differential attack. This is our next study work.

## 6. Conclusion

 A novel image encryption algorithm based on DNA subsequence operation is proposed in this paper. The simulation experimental results and security analysis show that the encryption algorithm is effective, easy to be realized, has larger key space, and is sensitive to the secret key. Our algorithm can also resist statistical analysis and exhaustive attacks. Furthermore, it avoids complex biological experiment in traditional DNA cryptography. But because DNA subsequence operation is based on horizontal, or the length of the subsequences selected is longer, it may lead to the horizontal correlation of the adjacent pixels in original image a bit high. We can improve the horizontal correlation through changing the lengths of DNA subsequences from each bit-planes. In addition to that, the weak ability of resisting differential attack is also a defect of this algorithm. They are our next research works.

## Figures and Tables

**Algorithm 1:**
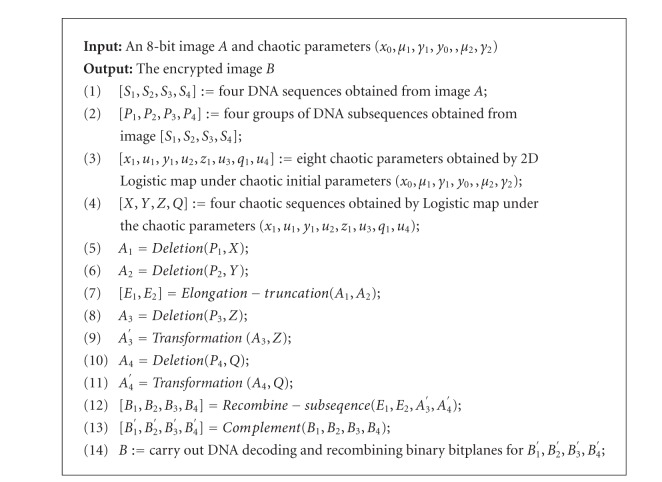
An image encryption algorithm based on DNA subsequence operation.

**Algorithm 2:**
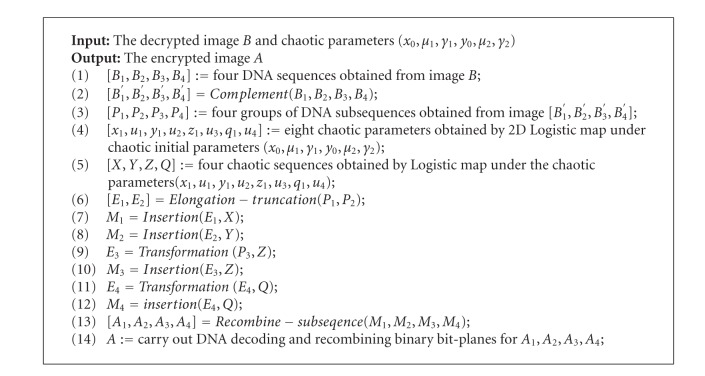
An image decryption algorithm based on DNA subsequence operation.

**Table 1:** Correlation coefficients of two adjacent pixels in two image.

Model	The original image	The encrypted image
Horizontal	0.9432	0.1366
Vertical	0.9688	0.0166
Diagonal	0.9148	0.0021

**Table 2:** Comparison with other chaos-based encryption algorithms.

Considered items for 256 × 256 lena image	Proposed	Reference [16]	Reference [17]	Reference [18]	Reference [1]
Key space	2^279^	2^233^	N/A	2^260^	2^99^
Key sensitivity	Yes	Yes	N/A	Yes	Yes
information entropy	7.9975	N/A	7.99732	7.9968	N/A

Chaotic system used	DNA operation and logistic system	Chen's chaotic system	Chen's chaotic system	Coupled chaotic system	Logistic chaotic system

**Table 3:** Comparison with other DNA-based encryption algorithms.

Considered items	Proposed	Clelland et al. [13]	Gehani et al. [14]	Kang [15]
Image	Yes	No	Yes	No
Text	Yes	Yes	Yes	Yes
Security analysis	Yes	No	No	Yes
Biology operation	No	Yes	Yes	No

Method	DNA subsequence operation chaotic maps	Messages encoded as DNA stands	Use micro-array technology	A pseudo DNA cryptography
